# Novel Lipid Nanocomplex Co-Carrying Bcl2 siRNA and Quantum Dots for EGF Receptor-Targeted Anti-Cancer Theranosis

**DOI:** 10.3390/ijms25116246

**Published:** 2024-06-06

**Authors:** Moon Jung Choi, Seong Jae Kang, Yeon Kyung Lee, Kang Chan Choi, Do Hyun Lee, Hwa Yeon Jeong, Min Woo Kim, Keun Sik Kim, Yong Serk Park

**Affiliations:** 1 Department of Medicine, Brown University, Providence, RI 02903, USA; moon_jung_choi@brown.edu; 2 Department of Biomedical Laboratory Science, Yonsei University, Wonju 26496, Republic of Korea; sjkang332@gmail.com (S.J.K.); yeonkyunglee80@gmail.com (Y.K.L.); dmadkr123@gmail.com (K.C.C.); dlehgus499@naver.com (D.H.L.); cca1987@korea.ac.kr (H.Y.J.); minwookim@yuhs.ac (M.W.K.); 3 Department of Biotechnology, College of Life Sciences and Biotechnology, Korea University, Seoul 02841, Republic of Korea; 4 Department of Surgery, Yonsei University College of Medicine, Seoul 03722, Republic of Korea; 5 Department of Biomedical Laboratory Science, Konyang University, Daejeon 35365, Republic of Korea; kskim11@konyang.ac.kr

**Keywords:** lipid micelle, nanomedicine, siRNA therapy, theranosis, quantum dot

## Abstract

Many different types of nanoparticles have been suggested for tumor-targeted theranosis. However, most systems were prepared through a series of complicated processes and could not even overcome the blood–immune barriers. For the accurate diagnosis and effective treatment of cancers, herein we suggested the lipid micellar structure capturing quantum dot (QD) for cancer theranosis. The QD/lipid micelles (QDMs) were prepared using a simple self-assembly procedure and then conjugated with anti-epidermal growth factor receptor (EGFR) antibodies for tumor targeting. As a therapeutic agent, Bcl2 siRNA-cholesterol conjugates were loaded on the surface of QDMs. The EGFR-directed QDMs containing Bcl2 siRNA, so-called immuno-QDM/siBcl2 (iQDM/siBcl2), exhibited the more effective delivery of QDs and siBcl2 to target human colorectal cancer cells in cultures as well as in mouse xenografts. The effective in vivo targeting of iQDM/siBcl2 resulted in a more enhanced therapeutic efficacy of siBcl2 to the target cancer in mice. Based on the results, anti-EGFR QDM capturing therapeutic siRNA could be suggested as an alternative modality for tumor-targeted theranosis.

## 1. Introduction

Progressed colorectal cancers are known to be highly fatal because of their high metastatic potential. They comprise a complex tumor fine environment consisting of various cancer-associated cells, and can become malignant in a short time through various growth factors [1]. Among these factors, the signaling pathway involving EGFR is directly linked to the oncogenesis and progression of colorectal cancers [2]. Based on these studies, the anti-EGFR antibody cetuximab has been FDA-approved for the treatment of cancers overexpressing the receptors [3]. Meanwhile, a great deal of effort has also been spent to utilize the EGF receptors as a target for therapeutic delivery [4,5,6].

One of the oncogenic causes of colorectal cancers is the overexpression of Bcl2 (B cell lymphoma 2), one of the sub-signal targets of the EGF receptor involved in the anti-apoptotic process [7]. The aberrant high expression of Bcl2 is associated with abnormal signaling via MEK1/2, BIM (BCL-2-interacting mediator of cell death), and Bax (BCL2 Associated X) downstream. Therefore, there have been various attempts to treat cancers through regulating the Bcl2 expression [8]. As one of the trials, the treatment with siRNA molecules to discontinue the expression of certain specific genes responsible for oncogenesis was suggested and has been clinically widely tested [9,10].

The siRNA binds to the sequence of the target mRNA by making a complex with RNA-induced silencing complex (RISC) proteins in the cytoplasm as the dsRNA of 15-25 base pair inhibits the expression of the target mRNA [11,12]. Various viral and lipid-based vectors have been developed to reliably deliver siRNA into the cell. But, many shortcomings such as inefficient delivery in vivo, immune cell recognition, and a lack of cell targetability have been resolved before clinical applications [13,14]. Therefore, various siRNA delivery systems have been conceptualized and tested for clinical translation.

Recently, within this field of research, the delivery vehicles which can carry anticancer therapeutics and diagnostic material simultaneously, so-called theranosis systems, have drawn serious attention in the field of nanomedicinal cancer therapy [15,16]. Various imaging agents have been proposed for diagnostics, and among them, QDs have gained prominence. With advantages such as diverse wavelength ranges, high imaging efficiency, and biocompatibility, QDs are actively researched as components of targeted tumor theranostic particles, alongside drug delivery vehicles [17,18]. It is essential to develop a reliable delivery nano system that can stably carry imaging agents and therapeutic drugs at the same time, efficiently delivering them to the intended cells or tissues. Already several different types of theranostic systems have been suggested, but the shortcomings mentioned earlier are still unavoidable [19,20]. There is an urgent need for the development of nanocarriers which can stably and efficiently deliver imaging agents and therapeutic drugs together.

Previously, we reported a tumor-targeted liposomal system carrying doxorubicin and QDs simultaneously, which could provide enhanced anticancer therapeutic efficacy in addition to target tumor imaging. In this study, we designed a novel tumor-targeted lipid micellar structure carrying hydrophobic QDs inside and siRNA exposed outward. This theranostic system was tested in the human colorectal cancer model in vitro and in vivo. The EGFR-directed micellar particles were small enough to efficiently move into the cancer tissues via the EPR (enhanced permeability and retention) effect, showing higher targetability and therapeutic efficiency when compared to the untargeted one. Based on these results, the tumor-targeted theranostic micellar structure can be clinically applicable to treat cancers as a delivery system platform carrying QDs and therapeutic siRNA. 

## 2. Results

### 2.1. Physiochemical Characteristics of QDMs

**Figure 1:**
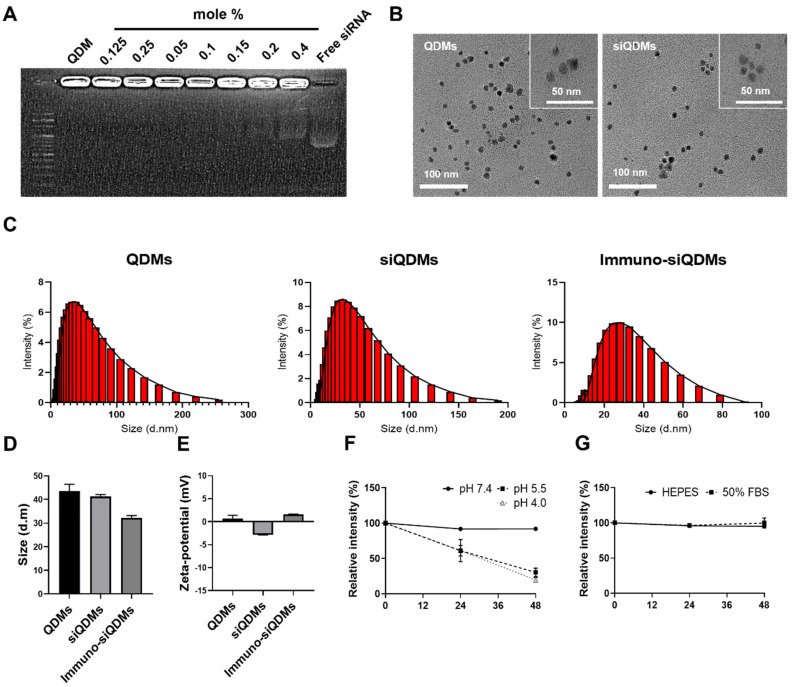
Characterization of the siRNA/QDs micelle (QDM/siRNA) complex. (**A**) Gel retardation assay results of QDM/siRNA. siRNA and QDM were incubated with various molar ratios. (**B**) Morphological property of QDM and QDM/siRNA was analyzed with TEM. (**C**,**D**) Size distribution of QDM, QDM/siRNA, and Immuno-QDM/siRNA. (**E**) Zeta−potential analysis of particles. (**F**,**G**) The pH and serum stability of QDM/siRNA were analyzed using the relative intensity of the release of QD from particles.

QDMs are solely composed of QDs, -PEG2000, and DSPE-PEG2000-maleimide lipid. The entire process of the preparation of QDMs was briefly described in the graphical abstract. Anti-tumoral bcl2 siRNA (siBcl2)-cholesterol conjugates (siBcl2-Chol) were incorporated into the preformed QDMs (QDM/siBcl2). At last, anti-EGFR antibody molecules were coupled to the maleimide moiety of the surface of QDM/siBcl2 (iQDM/siBcl2). Previously, we showed that bcl2 siRNA molecules complexed with cationic lipid nanoparticles were able to effectively reduce tumor growth in a mouse model [21]. Herein, we introduce a noble lipid micellar nanocarrier for anticancer siRNA molecules, as well as diagnostic QDs. siBcl2 molecules were able to be completely incorporated into QDMs at concentrations of 0.1 mole% or below (Figure 1A). A trace amount of siRNA molecules freed from the micelle structures was detected at 0.15 mole% or higher. 

According to the TEM pictures, QDMs and iQDMs showed homogeneous spherical structures, smaller than 50 nm in diameter (Figure 1B). The high mag pictures showed honeycomb shapes made of QD particles, which were surrounded by a monolayer of PEGylated phospholipids. The DLS analysis also showed a similar measurement. The measured diameters of QDMs, QDM/siBcl2, and iQDM/siBcl2 were 43.51 ± 5.08, 41.28 ± 1.34, and 32.13 ± 1.77 nm, respectively (Figure 1C,D). The surface charges of QDMs, QDM/siBcl2, and iQDM/siBcl2 showed 0.63 ± 1.30, −2.85 ± 0.05, and 1.56 ± 0.18, respectively (Figure 1E). All physicochemical properties of particles are described in Supplementary Table S1. As expected, siRNA-Chol insertion slightly reduced the zeta-potential of QDMs to negative values, but antibody conjugation increased the surface charges to positive values. However, the surface charges of the three QDMs can be considered as almost neutral, which may minimize the interactions with serum proteins during circulation in blood. QDMs smaller than 50 nm in diameter would be presumably beneficial for extravasation into tumor tissues in vivo via the EPR effect. 

To evaluate the pH and serum stability of iQDMs, iQDM/siBcl2 was incubated with varied pH conditions and 50% serum for 48 h. The destruction of iQDM integrity was analyzed via measurements of encapsulated quantum dot intensity. Under acidic conditions (pH 5.5 and 4.0), the iQDM/siBcl2 structure was slowly disintegrated, but the same particle integrity was stably maintained at pH 7.4 for 48 h (Figure 1F). Also, the nanostructure of iQDM/siBcl2 was stably maintained under the 50% serum condition (Figure 1G). These results imply that iQDM/siBcl2 would be stable enough to carry and deliver the cargo molecules, QD and siRNA, through the circulatory system in the body.

### 2.2. In Vitro Targetability of QDM/siBcl2

**Figure 2:**
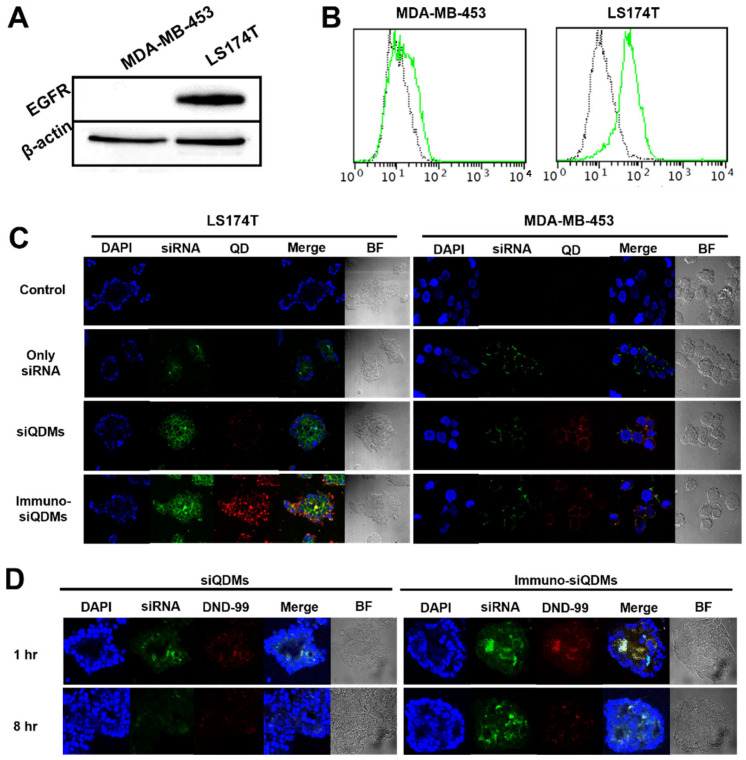
Tumor-targeted siRNA delivery of QDM and immuno-QDM. Protein expression of MDA-MB-453 (EGFR negative) and LS174T (EGFR positive) cells, and the target tumor cell binding and cellular uptake assay of Chol-siRNA, QDM, and immuno-QDM (**A**–**C**) were evaluated. All particles were incubated with the target cells (LS174T) for 1 h and 8 h at 37 °C and analyzed with a confocal microscope for evaluate siRNA delivery and endosomal escape analysis of QDM and immuno-QDM (**D**) (X400).

For the evaluation of the tumor targetability of iQDMs, two different cell lines, MDA-MB-453 as EGFR-negative and LS174T as EGFR-positive one, were used. The expression of EGF receptors was verified using Western blotting (WB) and FACS analysis before tumor targetability assays (Figure 2A,B). LS174T showed high expression of EGFR, but the MDA-MB-453 showed no expression in WB results. And also EGFR expression level was anaylzed with FACS using anti-human EGFR antibody (black: non-labeled control, green: PE-anti-EGFR). To compare the delivery of siRNA and QDs with QDMs and iQDMs, target LS174T cells and control MDA-MB-453 cells were treated with QDMs or iQDMs with FITC-labeled siBcl2 for 1 h at 37 °C. According to the microscopic analyses, MDA-MB-453 cells were overall less susceptible to the transfection system than the target LS174T cells under the same transfection conditions. Nevertheless, the anti-EGFR iQDM/siBcl2 did not show any enhanced delivery of QDs (red) or siRNA (green) to MDA-MB-453, just similar to the bare QDM/siBcl2 (Figure 2C). Meanwhile, the iQDM/siBcl2 exhibited greater delivery of QDs and siRNA to the target LS17T4 cells than the bare QDM/siBcl2. These data clearly show that targeting antibodies against the EGF receptors are able to enhance the delivery of therapeutic siRNA molecules and diagnostic QDs at the same time. 

The anti-EGFR micelles appeared to be endocytosed via EGR receptors. To verify the endocytic process, the iQDMs containing FITC-siRNA and DND-99 lysotracker were detected using the confocal microscope 1 and 8 h after treatment. One hour after treatment, the treated LS174T cells were more susceptible to iQDMs than the bare QDMs, and most delivered siRNA molecules were co-localized with DND-99 in endosomal vesicles. However, 7 h later, the colocalization was diminished, and, at the same time, the siRNA molecules carried by iQDMs were seen throughout the cytoplasm (Figure 2D). The efficnet release of siRNA from the micelles and the escape from the endosomal process are crucial for target gene silencing. QDMs and iQDMs were treated to LS174T cells for 8 h and detected at 1 h and 8 h. According to these results, the anti-EGFR antibodies exposed on the carriers enhanced the target cell recognition. The siRNA molecules could be stably carried by the micellar carriers and then released into the cytoplasm.

### 2.3. Interference of In Vitro bcl2 Expression by iQDM/siBcl2

**Figure 3:**
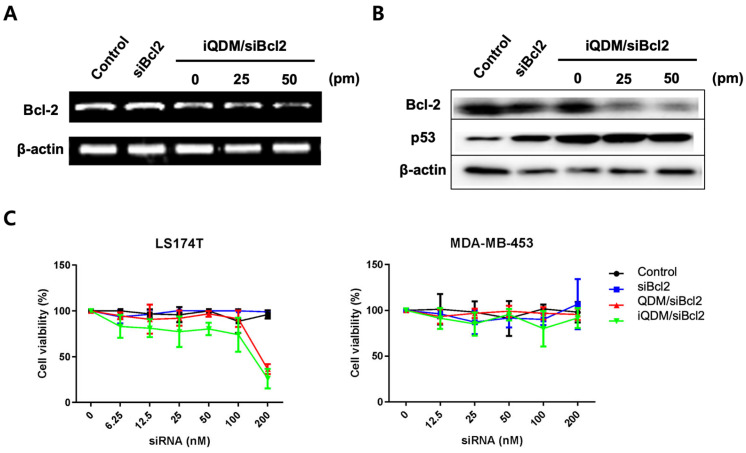
Knockdown of target gene and in vitro anti-cancer efficacy of QDM/siBcl2 and iQDM/siBcl2. (**A**,**B**) The bcl2 gene knockdown was detected with PCR and Western blot (25, 50 pm). (**C**) MTT assay results of LS174T and MDA-MB-453 cells after the treatment of siRNA, QDM/siBcl2, and iQDM/siBcl2.

To assess the downregulation of bcl2 expression mediated by iQDM/siBcl2, LS174T cells were subjected to varying concentrations of iQDM/siBcl2 (0−200 picomoles of siRNA) for a duration of 24 h. The expression of bcl2 was examined using a quantitative polymerase chain reaction (qPCR) and Western blot analysis. In comparison to cells treated with free siRNA, those treated with iQDM exhibited a dose-dependent decrease in bcl2 mRNA levels in vitro (refer to Figure 3A). Treatment with 50 picomoles of iQDM/siBcl2 leads to a notable decrease (approximately 60–70%) in the bcl2 expression levels when compared to only the vehicle (0 picomole). Additionally, higher concentrations of iQDM/siBcl2 demonstrate a dose-dependent inhibition of bcl2 expression. Cells treated with concentrations exceeding 50 picomoles of siRNA delivered via iQDMs exhibit a significant reduction in bcl2 protein expression. These findings suggest a correlation between the treatment concentration and the efficacy of bcl2 downregulation, highlighting the potential of iQDM/siBcl2 as a promising therapeutic approach for modulating bcl2 expression levels (see Figure 3B). 

To measure the in vitro cytotoxicity of iQDM/siBcl2, various concentrations of iQDMs containing siBcl2 were administered to target LS174T and control MDA-MB-453 cells. According to the MTT analysis (Figure 3C), the cell viability of LS174T began to show intergroup differences at 100 nM. The free siRNA treatment, QDM, and iQDM groups exhibited mean viabilities of 88.75%, 91.4%, and 73.97%, respectively. A distinct difference was observed at a concentration of 200 nM, with mean viabilities of 96%, 36.45%, and 26% in the respective groups. The viability of control MDA-MB-453 cells treated with the same concentration did not show significant changes. These results also indicate that EGFR-targeted lipid micelles were able to deliver siBcl2 to the target cells, and the delivered siBcl2 molecules effectively interfered with bcl2 expression in the target cells, resulting in reduced viability.

### 2.4. In Vivo Biodistribution of iQDM/siBcl2

**Figure 4:**
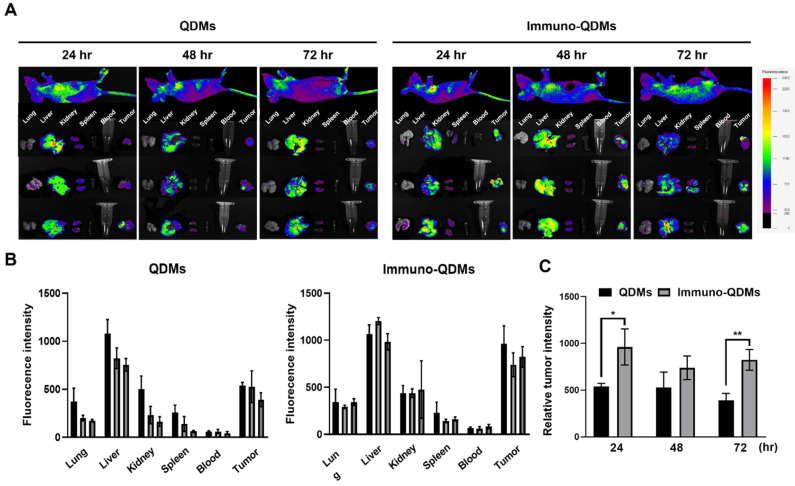
In vivo tumor and major organ biodistribution of QDMs and immune-QDMs in tumor xenograft model. (**A**,**B**) QDMs and immuno-QDMs were injected into tumor xenograft mice (*n* = 3) and a QD signal was detected with a NEO in vivo imaging system for 72 h. (**C**) The accumulation of the tumor signal of QD was analyzed at all time points. Each value represents the mean ± S.D. for three separate experiments. * *p* < 0.05, and ** *p* < 0.01 between experimental groups.

To evaluate the in vivo biodistribution of iQDMs, the QDM/siBcl2 or iQDM/siBcl2 was intravenously injected into the mice xenografted with LS174T colon cancer. The tumor bioluminescence image is obtained using a QD signal. The ex vivo bioimaging of major mouse organs was taken 72 h after injection. According to the fluorescence imaging given by QDs in the micelles, the tumor-targeting iQDMs exhibited a higher accumulation in the tumor tissues than the nontargeting QDMs across the all time points measured (Figure 4A). As expected, the liver was still the major organ taking up the QDMs and iQDMs (Figure 4B). At 72 h post-injection, the QD signals of the liver reduced time-dependently, but a signal of tumors was not reduced in the iQDMs group compared to the 48 h (Figure 4C). These results demonstrated that antibody conjugation enhanced the tumor targetability and accumulation of particles. Also, QDs could be applied for diagnostic molecules in cancer imaging. 

### 2.5. In Vivo Anti-Tumor Therapeutic Efficacy of iQDM/siBcl2s

**Figure 5:**
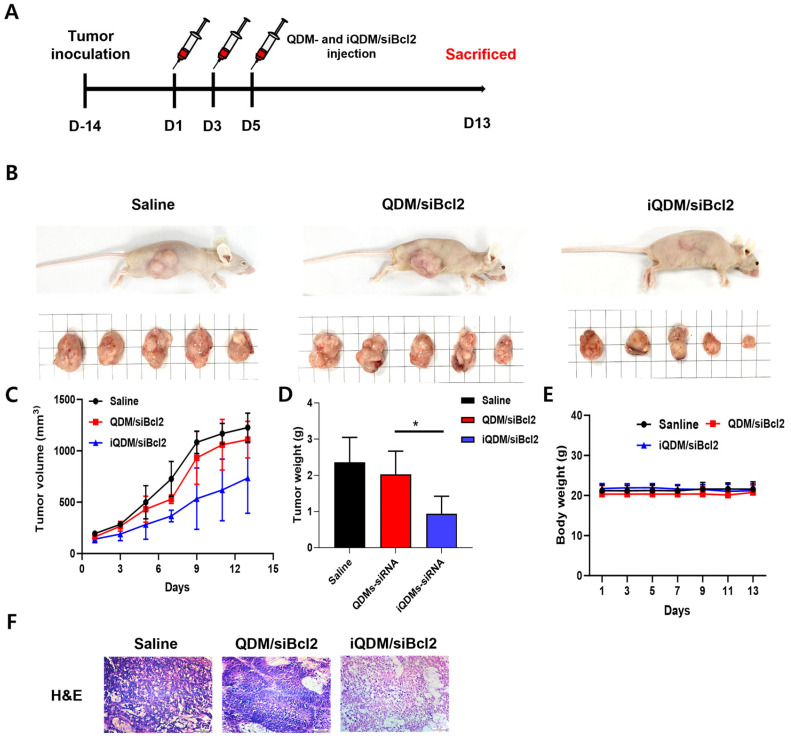
In vivo tumor growth inhibition of QDM/siBcl2 and iQDM/siBcl2. All mice were subcutaneously injected with 1 × 10^7^ tumor cells (n = 5). And each mice group was intravenously injected with saline, QDM/siBcl2, and iQDM/siBcl2. (**A**) Images of tumor-bearing mice and dissected tumor tissues. (**B**) Tumor volume and body weight of mice were reported for 17 days after injection. (**C**–**E**) Tumor weight of mice was measured at the last time point. (**F**) The HE stain results of the tumor tissues of all experimental groups. Each value represents the mean ± S.D. for three separate experiments. * p < 0.05 between experimental groups. Each scale bar represents 100 µm.

**Figure 6:**
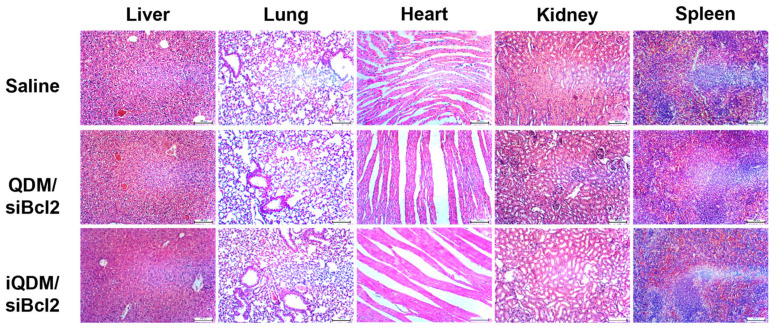
Histopathological analysis of major organs of all mice experimental groups. Major organs, including the heart, lungs, spleen, and kidneys, were dissected from the treated mice on day 13 after the administration of various micelle formulations. The paraffin-embedded tissue sections of the organs were stained with HE and observed under a light microscope. Magnification: ×100.

The in vivo anti-tumor effect of QDM/siBcl2 and iQDM/siBcl2 was evaluated with a LS174T colorectal cancer xenograft of a mouse. Mice were injected with saline, QDM/siBcl2, and iQDM/siBcl2 via iv injection every two days for a total of three times. The tumor volume and body weight of the mice were measured twice a week (Figure 5A). All mice were sacrificed on day 13 after starting the experiment (D0). In vivo tumor-bearing mice and dissected tumors are represented in Figure 5B. As shown in Figure 5C, iQDM/siBcl2 exhibited the most effective tumor growth inhibition. Also, the tumor weight results reflected the tumor volume (Figure 5D). No change was observed in all experimental groups (Figure 5E). The tumor tissue treated with iQDM/siBcl2 showed many parts of necrosis, but the saline groups exhibited the aggressive cancer cells (Figure 5F). The immunohistochemistry analysis demonstrated that major organs treated with QDM/siBcl2 and iQDM/siBcl2 showed no cellular toxicity via HE staining (Figure 6). These results highlight that iQDMs stably delivered siBcl2, and iQDM/siBcl2 exhibited the efficient target therapy of colorectal cancer.

## 3. Discussion

In our investigation, we have successfully engineered a pioneering micellar theranostic system known as iQDMs. This advanced delivery platform is meticulously crafted from a simplistic blend of phospholipids intricately combined with tumor-targeting antibodies. The smaller size of these particles, in comparison to conventional nanoparticles, is expected to exhibit more effective drug delivery in a sophisticated colorectal cancer model representing the tumor microenvironment. Superior drug delivery efficacy is anticipated not only in other cancers with insufficient angiogenesis, but also in small-sized tumors. Notably, iQDMs demonstrate a remarkable ability to encapsulate quantum dots (QDs) and small interfering RNA (siRNA) with exceptional stability and efficacy. Through the encapsulation of siRNA within the micellar structure, we have achieved a reliable means of delivering therapeutic agents to the cytoplasm of cancer cells, thereby facilitating sustained anti-tumor effects. Moreover, the encapsulation efficiency of both QDs and siRNA reached sufficient levels to elicit potent anti-cancer responses. The prominent advantages of the particles lie in their ability for self-assembly, enabling simple fabrication processes and ensuring high stability. These attributes not only enhance biocompatibility, but also result in a longer lifespan when compared to other particles. Over a span of 72 h post-injection, iQDMs were observed to accumulate within tumor tissues, underscoring their potential utility in tumor imaging utilizing QDs, while concurrently affirming the stability of QDs as diagnostic agents. It is particularly noteworthy that, while the accumulation of anticancer genes decreased over time in particles lacking tumor targeting, iQDMs continued to deliver genes to cells even after up to 8 h, maintaining this delivery efficacy. Additionally, the strategic process of antibody conjugation to the micelles synergistically contributed to enhancing the tumor-targeting capabilities. Furthermore, comprehensive immunohistochemical analyses have unequivocally demonstrated the absence of any discernible toxicity associated with iQDMs across major organs. This is indicative of their superior safety profile. Despite their very simple composition when compared to other particles, the particles demonstrated high stability and low biotoxicity, based on enhanced gene delivery efficiency and remarkable gene escape ability. This robust safety profile, coupled with the inherent attributes of iQDMs, including their stable structure, unparalleled encapsulation efficiency, and enhanced tumor-targeting precision, positions them as highly promising contenders for a myriad of gene delivery applications. In addition to the demonstrated efficacy in colorectal cancer models, the versatility of iQDMs suggests potential applications across a broad spectrum of cancers. For instance, in tumors characterized by dense stromal environments or those with poor vascularization, the small size and targeting ability of iQDMs can facilitate the deeper penetration and more efficient delivery of therapeutic agents. This could lead to significant advancements in the treatment of notoriously hard-to-treat cancers such as pancreatic and brain tumors. Moreover, the unique properties of iQDMs open new avenues for personalized medicine. The ability to conjugate specific antibodies to the micellar surface allows for the customization of the particles to target unique tumor antigens, thereby enhancing the specificity and reducing of off-target effects. This personalized approach could revolutionize cancer therapy, providing tailored treatments that improve patient outcomes while minimizing the side effects. In conclusion, the development of iQDMs represents a significant advancement in the field of nanomedicine. Their innovative design, combining simplicity and sophistication, offers a potent platform for targeted drug delivery and diagnostic imaging. As we continue to explore and refine these micellar systems, their potential to transform cancer treatment and improve patient outcomes becomes increasingly evident. Future studies should focus on clinical translation and exploring the full range of applications of iQDMs in cancer therapy and beyond.

## 4. Materials and Methods

### 4.1. Materials

For this experiment, 1,2-distearoyl-sn-glycero-3-phosphoethanolamine-N-[amino(polyethylene glycol)-2000] (DSPE-PEG2000-amine) and 1,2-distearoyl-sn-glycero-3-phosphoethanolamine-N-[maleimide(polyethylene glycol)2000] (DSPE-PEG2000-Mal) were purchased from Avanti Polar Lipid, Inc. (Alabaster, AL, USA). AccuTarget™ fluorescein-labeled scrambled siRNA (SN-1023), AccuTarget™ scrambled siRNA (SN-1003), and siBcl2-Chol (No.1011940) were purchased from Bioneer Inc. (Daejeon, Republic of Korea). CdSe/ZnS high-quality organic NSQ-quantum dots (Q-dots, λ_emit_ = 620 nm) were purchased from Nanosquare Inc. (Seoul, Korea). Paclitaxel was purchased from Sigma-Aldrich (St. Louis, MO, USA). The anti-EGFR antibody cetuximab was purchased from Merck KGaA (Darmstadt, Germany). 

### 4.2. Animals

Six-week-old female BALB/c nude mice were purchased from Nara Biotech (Seoul, Republic of Korea). All animal experiments were approved by the Institutional Animal Care and Use Committee (IACUC) of Yonsei University, Mirae Campus (YWCI-201706-011-03) and were performed according to the school guidelines and regulations.

### 4.3. Cell Lines and Cell Culture

The LS174T (human colorectal adenocarcinoma, #10188) cell line was purchased from the Korean Cell Line Bank (Seoul, Republic of Korea). The MDA-MB-453 (human breast metastatic carcinoma, HTB-131™) cell line was purchased from the American Type Culture Collection (Manassas, VA, USA). LS174T cells were maintained in RPMI 1640 (Gibco, Carlsbad, CA, USA) supplemented with 10% fetal bovine serum (FBS, Gibco), 100 IU/mL penicillin (Gibco), and 100 μg/mL streptomycin (Gibco) under a humidified atmosphere of 95% air and 5% CO_2_ at 37 °C. The MDA-MB-453 cells were maintained in Leibovitz’s L-15 medium (Welgene, Gyeongsan, Republic of Korea) supplemented with 10% FBS (Gibco), 100 IU/mL penicillin (Gibco), and 100 μg/mL streptomycin (Gibco) under a CO_2_-free humidified atmosphere at 37 °C.

### 4.4. Synthesis of Micelles Encapsulating QDs (QDMs)

QDs were encapsulated in lipid micelle cores via the hydration of the dried lipid thin film. First, DSPE-PEG_2000_-amine in a chloroform/methanol solution (2:1, *v*/*v*) and QDs in chloroform were mixed at a molar ratio of 700:1. The organic solvents were then evaporated under a stream of N2 gas. Vacuum desiccation was performed fir 1 h to remove the residual organic solvents. The dried film was incubated at 80 °C for 2 h and then hydrated with HEPES buffer (25 mM HEPES, 140 mM NaCl, pH 7.4; 1 mg lipid per mL). Uncaptured QD aggregates were removed via filtering through a Nuclepore Track-Etched polycarbonate membrane filter (Whatman Inc., Piscataway, NJ, USA) with a 100 nm pore size using the extrusion method. Insert a 100 μm polycarbonate filter membrane into the stainless steel filter holder of the extruder. Next, place the extruder heating block onto a hot plate. Turn on the hot plate and set its temperature to exceed the transition temperature (Tc) of the lipids. Once the desired temperature is reached, re-wet the extruder components by passing a syringe filled with buffer through the extruder. Remove the buffer afterward to reduce the dead volume. Fill one 1 mL Hamilton syringe with a solution containing quantum dots, attach the needle to the holder, and secure an empty Hamilton syringe to the other side. Slowly alternate between the two syringes to extrude the solution. This process will filter out quantum dot aggregates larger than 100 nm, as they will be trapped in the membrane. The concentrations of QDs were estimated via measuring the absorbance at 310 nm using an Infinite 200 Pro NanoQuant (TECAN Group Ltd., Mannedorf, Switzerland). The QD-encapsulating micelles were referred to as QDMs.

### 4.5. Conjugation of the Anti-EGFR Antibody to the Surface of the QDMs

To couple anti-EGFR antibodies to the surface of QDMs, 1 mol% of DSPE-PEG_2000_-Mal in a chloroform/methanol (2:1, *v*/*v*) solution was added to the DSPE- PEG_2000_-amine and QDs mixture in the organic solvent. QDMs containing 1 mol% of DSPE-PEG_2000_-Mal, referred to as the QDM-Mal, were prepared as described above. Primary amine groups of cetuximab (25 mM HEPES, 140 mM NaCl, 2 mM EDTA, pH 8.0) were thiolated via treatment with a 10-fold molar excess of Traut’s reagent (Thermos Scientific, Rockford, IL, USA) in the same buffer for 1 h at RT. The reaction mixture was then passed through a PD-10 desalting column (GE Healthcare, Chicago, IL, USA) to remove unreacted Traut’s reagent. The thiolated antibody was quantified using the Bradford protein assay kit (Bio-Rad, Hercules, CA, USA). The thiolated antibody was added to the QDM-Mal at a 0.2:1 molar ratio of antibody and DSPE-PEG_2000_-Mal, and the mixture was then incubated for 18 h at 4 °C (Supplementary Figure S1). The antibody conjugation was verified through Coomassie blue staining and ultraviolet light illumination following 10% sodium dodecyl sulfate-polyacrylamide gel electrophoresis (SDS-PAGE). The antibody conjugation efficiency was estimated through measuring the density of bands stained with Fusion Solo Chemidoc (Viler Doormat, Eberhard Zell, Germany). The QD-encapsulating micelles were also examined under UV illumination. Unconjugated antibodies were removed by centrifugation using a microfuge membrane filter (NANOSEP 100 K OMEGA, Pall Corporation, New York, NY, USA) at 14,000× *g* for 15 min. The anti-EGFR antibody-conjugated QDMs were referred to as anti-EGFR immuno-QDMs (iQDMs). Finally, to incorporate siRNA, siRNA-cholesterol conjugates (0.1 mole%) were added to QDMs or iQDMs and then vigorously mixed with a vortex mixer for 20 min at RT. The QDMs containing Bcl2 siRNA were referred to as QDM/siBcl2.

### 4.6. Analyses of QDMs 

The physicochemical properties of QDMs such as particle sizes and surface charges were measured thrice via dynamic light scattering (DLS) analysis with Zetasizer Nano-ZS90 (Malvern Instrument Ltd., Malvern, UK). The measurements were statistically analyzed using the GraphPad Prism software 8.2.1 (GraphPad Software, Inc., San Diego, CA, USA). The QDMs were also viewed under a transmission electron microscopy. An aliquot of the QDMs and iQDMs solution (4 μL of 1 mg lipid per mL) was placed on a carbonyl-coated 400 mesh copper grid (CF400-CU-UL, Electron Microscopy Sciences, Hatfield, PA, USA) for 15 min. The solution was removed via gentle tapping with a piece of filter paper and then dried out for 10 min at room temperature. For negative staining, 10 μL of 2% uranyl acetate was placed on the grid for 10 min, removed, and dried as described above. The negatively stained micelles on the grid were observed with an electron microscope (JEM-2100F, JEOL Ltd., Tokyo, Japan) at magnifications of 80 K and 150 K.

### 4.7. Analysis of Bcl2 siRNA Capturing in QDMs 

Gel retardation assays were performed to prove that Bcl2 siRNA (siBcl2) was being captured to the preformed QDMs. The varied mole% of siRNA-cholesterol (0.0125 to 0.4) were mixed with preformed QDMs as described earlier. The samples were mixed with 6× loading dye and then loaded on a 1.5% agarose gel prepared in 0.5× Tris/borate/EDTA (TBE) buffer. The samples were run at 100 V for 20 min and visualized using the Quantity One program of the Gel Doc EQ system (Bio-Rad Lab., Hercules, CA, USA). 

### 4.8. pH and Serum Stability of QDMs

QDMs were incubated at three different pH levels (pH4, 5.5, and 7.4) for 24 or 48 h, and then the QD intensity of intact QDMs was compared via measuring the absorbance at 310 nm with an Infinite 200 Pro NanoQuant (TECAN Group Ltd.). To evaluate the QDM stability in the presence of serum, an aliquot of QDMs was added to FBS (1:1, *v*/*v*) and then incubated for 24 or 48 h at 37 °C. The QD intensity of intact QDMs in the FBS solution was also measured after incubation.

### 4.9. Analyses of Target Cell Binding and Cellular Uptake of QDMs

For the cell binding analysis, QDM/siBcl2 and iQDM/siBcl2 were treated with EGFR-positive LS174T cells and EGFR-negative MDA-MB-453 cells (both 5 × 10^5^ cells in 300 μL per tube) for 1 h at 4 °C. The treated cells were washed twice with PBS (pH 7.4) containing 0.1% bovine serum albumin (BSA). The lipid micelles bound to the cancer cells were analyzed using FACS Calibur flow cytometer (BD Biosciences, San Jose, CA, USA) and CellQuest software version 3.3 (BD Biosciences).

For the cellular uptake analysis, LS174T and MDA-MB-453 cells (both 5 × 10^5^ cells/well) were seeded on coverslips in 6-well plates and cultured for 24 h. The QDMs or iQDMs containing FITC-labeled siBcl2 were added to the cells and then incubated for 1 h at 37 °C in a serum-free medium. After incubation, the cells were washed twice with cold PBS and fixed with 2% paraformaldehyde for 10 min at 4 °C. The cells were stained with one drop of 4′,6-diamidino-2-phenylindole (DAPI) solution (Vector Lab, Burlingame, CA, USA) for 10 min in the dark and then mounted onto slides. The slides were observed using a confocal laser scanning microscope (LSM 510; Zeiss, Heidenheim, Germany). For intracellular localization analysis, the cancer cells were treated with QDMs and iQDMs containing FITC-siRNA (50 pmol) and then incubated at 37 °C for varied periods of time. The dye for tracking endocytosis, Lysotracker Red DND-99 (100 nM; Invitrogen, Carlsbad, CA, USA), was added to each well and then incubated for 1 h. The treated cells were observed using a confocal laser scanning microscope as described above.

### 4.10. In Vitro siRNA Transfection of iQDMs

EGFR positive LS174T cells were seeded in 6-well plates (5 × 10^5^ cells/well) and transfected with iQDMs encapsulating bcl2 siRNA in a serum-free medium for 4 h and then further incubated in a medium with 10% FBS for 24 h. Total RNA was isolated from LS174T cells using Trizol reagent (Invitrogen) according to the manufacturer’s instructions. The cDNA was then synthesized via reverse transcription with 2 μg of the isolated RNA, and the subsequent reverse transcription polymerase chain reaction (RT-PCR) using the reverse transcription master premix (dT, 5×, M-MLV-RT, RNase H+) (ELPIS Biotech, Daejeon, Republic of Korea) was performed in a thermocycler according to the manufacturer’s recommendations. PCR products were electrophoresed on 2% agarose gels prepared in TBE buffer, stained for 10 min with ethidium bromide (EtBr), and de-stained for 20 min in tap water. Gel images were captured using a Gel Doc EQ system (Bio-Rad Laboratories, Hercules, CA, USA).

### 4.11. Western Blotting

The knockdown of the target gene by bcl2 siRNA transfection was analyzed via Western blotting. The transfected cells were lysed in RIPA buffer (Sigma-Aldrich) with Halt™ protease inhibitor (Thermo Scientific, Boston, MA, USA). The lysate was centrifuged at 19,000× *g* for 10 min at 4 °C. The total concentration of cellular proteins was determined using a BSA protein standard kit (Bio-rad). The protein samples were electrophoresed on 10% PAGE gel (120 V, 1.5 h), and then transferred to nitrocellulose blotting membranes (Pall Corp., Port Washington, NY, USA) for 1 h at 350 mA. Protein blots were detected by SuperSignal^®^ West Pico (Thermo Scientific) or Dogen EZ-Western Lumi Femto (Daeil Lab Service Co. Ltd., Seoul, Republic of Korea) using Fusion Solo Chemidoc (Vilber Lourmat, Paris, France).

### 4.12. Evaluation of Particle Cytotoxicity 

The in vitro cytotoxicity of QDM/siBcl2 and iQDM/siBcl2 was measured using the Cell Counting Kit-8 (CCK-8, Dojindo Laboratories, Kumamoto, Japan). LS174T and MDA-MB-453 cells were seeded into 96-well plates (1 × 10^4^ cells/well). The cells were treated with QDMs in a serum-free medium for 4 h and then further incubated with the medium containing 10% FBS for 48 h. After incubation, 10 μL of CCK-8 solution was added to the cells, which were incubated for another 2 h. The absorbance at 450 nm was measured with the Infinite 200 Pro NanoQuant.

### 4.13. Animal Studies

All animal experiments were approved by the Institutional Animal Care and Use Committee (IACUC) of Yonsei University at Wonju (YWCI-202005-N-002-02), and were performed according to their guidelines and regulations. To prepare tumor xenografts, 6-week-old female BALB/c nude mice (Narabiotech, Seoul, Republic of Korea) were subcutaneously inoculated with LS174T cells in saline (1 × 10^7^ in 200 μL) at the 4th mammary fat pad. Tumor volumes were measured with calipers and calculated using the following equation: tumor volume = length × width^2^/2. When the tumors grew to ~150 mm^3^, the mice were injected with the QDM/siBcl2 and iQDM/Bcl2 via the tail vein (10 mg lipid/kg). For the analysis of QDM biodistribution, the treated mice were placed in a prone position on the bed and kept under anesthesia with 2.5% isoflurane. The whole body fluorescence images of tumors were acquired using the FOBI in vivo imaging system (CELLGENTEK, Deajeon, Korea) at 24, 48, and 72 h post-injection. After taking the fluorescence images, all the mice were immediately sacrificed and major organs including tumors were then dissected. The fluorescence intensities of the organs were measured using the FOBI imaging system. For the analysis of the tumor growth inhibition, the mice with LS174T tumor xenografts were randomly divided into 6 groups (n = 5). The mice were intravenously administered with saline, QDM/siBcl2, and iQDM/siBcl2 (200 pm siRNA per mouse) 3 times with 2 day intervals. The tumor volumes and body weights of the mice were measured two times a week. All mice were sacrificed on day 13 after treatment, and the tumor weight and dimensions were measured. To show any in vivo toxicity of the QDMs, major organs were resected from the sacrificed mice. The organs were formalin-fixed, embedded in paraffin, and sectioned into 6 μm. The tissue sections were stained with hematoxylin and eosin (HE), and the tissue slides were photographed using optical microscopy (Leica, Wetzlar, Germany) and rendered using Leica software (LASX Office 1.4.6).

### 4.14. Statistical Analysis

Data are represented as mean ± standard deviation (S.D.). Statistical analysis was performed with one-way or two-way ANOVA using Prism 8.4 (GraphPad Software, Inc., La Jolla, CA, USA). * *p* < 0.05, ** *p* < 0.01 and *** *p* < 0.001 vs. control or between experimental groups. *p* < 0.05 between experimental groups.

## 5. Conclusions

Based on these findings, the micellar nanoparticles developed in this study, consisting of lipids and quantum dots as imaging materials, have been optimized for siRNA delivery. It is anticipated that these particles can also be used for delivering various therapeutic genes, including miRNA, mRNA, and pDNA, in research. Additionally, the utilization of various types of quantum dots suggests their potential as advanced diagnostic materials.

## Data Availability

The data presented in this project are available in this article.

## Supplementary Materials

The following supporting information can be downloaded at https://www.mdpi.com/article/10.3390/ijms25116246/s1.

## Abbreviations

QD	Quantum dot
QDM	QD/lipid micelle
EGFR	Epidermal growth factor receptor
RISC	RNA-induced silencing complex
EPR	Enhanced permeability retention
SDS-PAGE	Sodium dodecyl sulfate-polyacrylamide gel electrophoresis
DLS	Dynamic light scattering
BSA	Bovine serum albumin
DAPI	4′,6-diamidino-2-phenylindole
qPCR	Quantitative polymerase chain reaction
FDA	Food and drug administration
FITC	Fluorescein isothiocyanate
HE	Hematoxylin and eosin
TUNEL	Terminal deoxynucleotidyl transferase dUTP nick end labeling
