# Supplementary material for: Novel Lipid Nanocomplex Co-Carrying Bcl2 siRNA and Quantum Dots for EGF Receptor-Targeted Anti-Cancer Theranosis
Source: Int J Mol Sci. 2024 Jun 6;25(11):6246. doi: 10.3390/ijms25116246 (PMC11172456; doi:10.3390/ijms25116246)
Supplement: Supplementary file 1 [file ijms-25-06246-s001.zip › ijms-3017713-supplementary.pdf]

Supplementary Table S1. Physicochemical properties of various micellular particles.

Particles	Size (nm)*	Zeta-Potential (mV)*	Polydispersity Index
**QDMs**	43.51 ± 5.08**	0.633 ± 1.30**	0.05
**QDMs-siRNA**	41.28 ± 1.34	-2.850 ± 0.05	0.12
**iQDMs-siRNA**	32.13 ± 1.77	1.560 ± 0.18	0.17

* The particle size and zeta-potentials were analyzed three times using a zeta-sizer.

** The particle size (nm); average particle size ± S.D. Zeta potential (mV); average zeta-potential ± S.D.

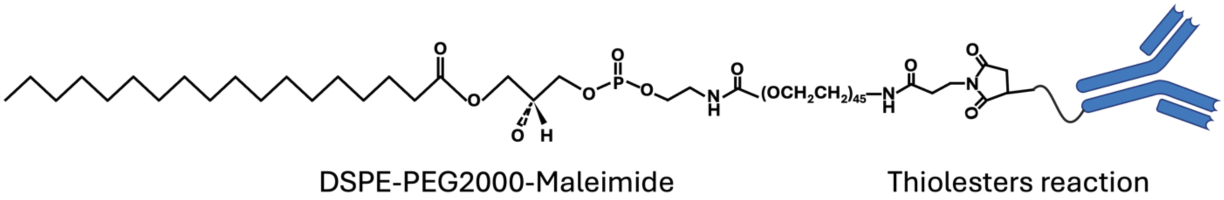

Supplementary figure S1. Conjugation of lipid composed iQDMs with thiolated antibody.
DSPE-PEG2000-Maleimide was conjugated with thiolated anti-EGFR antibody for iQDM.
